# Programmed Cell Death in Heart Failure: Mechanisms, Impacts, and Therapeutic Prospects

**DOI:** 10.31083/RCM38407

**Published:** 2025-07-28

**Authors:** Dongda Wu, Donghong Deng, Biao Tang

**Affiliations:** ^1^Medical School, Hunan University of Chinese Medicine, 410208 Changsha, Hunan, China; ^2^People’s Hospital of Ningxiang City, Hunan University of Chinese Medicine, 410600 Changsha, Hunan, China

**Keywords:** heart failure, programmed cell death, apoptosis, autophagy, necroptosis, pyroptosis, ferroptosis

## Abstract

Heart failure is a complex pathological condition characterized by various mechanisms of cellular death, among which programmed cell death (PCD) plays a crucial role in the pathophysiology of cardiac dysfunction. This review delves into the different forms of PCD present in heart failure, including apoptosis, autophagy, necroptosis, pyroptosis, and ferroptosis, and examines the mechanisms of action involved and the potential therapeutic targets for treating cardiac failure. By analyzing the latest research findings, we reveal the pivotal role of PCD in the progression of heart failure and discuss the preclinical prospects of intervening in these processes to develop novel therapeutic strategies. For instance, pharmacological agents that inhibit receptor-interacting protein kinases (RIPK1 and RIPK3) involved in necroptosis have been demonstrated to reduce cardiac injury and improve functional outcomes. Additionally, targeting the inflammatory responses associated with necrotic cell death, such as using interleukin (IL)-1β inhibitors, may provide a dual benefit by reducing cell death and inflammation. Thus, combining current knowledge will enhance our understanding in this field and promote innovative approaches to managing heart failure more effectively.

## 1. Introduction

Heart failure (HF) is a major global health concern. Its incidence and mortality rates have been steadily increasing. This condition stems from several underlying heart diseases that impair cardiac function. Recent studies have highlighted the significant role of programmed cell death (PCD) in the development and progression of heart failure. PCD includes several pathways that facilitate the regulated elimination of cells, such as apoptosis, autophagy, necroptosis, pyroptosis, and ferroptosis, all of which are crucial for maintaining healthy tissue balance [1]. In heart failure, the loss of cardiomyocytes through these PCD mechanisms is a key factor in the progression of the disease, contributing to adverse changes in heart structure and function [2]. For example, processes like apoptosis, necroptosis, pyroptosis, and ferroptosis can be activated by various stress factors, including ischemia and oxidative stress, which are common in heart failure. These processes not only compromise the structural and functional integrity of heart cells but also have a strong correlation with patient outcomes [3, 4]. Autophagy, a process that breaks down cellular components, has a complex role in heart failure. It can support cell survival under stress but, when dysregulated, can lead to cell death and worsen cardiac function [5].

Emerging evidence suggests that the interplay between these cell death pathways is complex. Understanding the mechanisms of these cell death processes and their interplay is essential for developing innovative therapeutic strategies aimed at improving the treatment of heart failure and improving patient survival. Understanding the molecular pathophysiology of these processes may lead to the identification of novel biomarkers for prognosis and therapeutic targets for intervention. Gene therapy and cell therapy represent the forefront of innovative treatment strategies with the potential to revolutionize heart failure management. Gene therapy interventions, particularly those utilizing Clustered Regularly Interspaced Short Palindromic Repeats/CRISPR-associated protein 9 (CRISPR/Cas9) technology, are being explored for their ability to correct genetic defects associated with heart failure and to enhance the regenerative capacity of cardiac tissues. Recent advancements in gene editing have shown promise in addressing inherited forms of heart disease, such as hypertrophic cardiomyopathy and dilated cardiomyopathy, by targeting specific mutations that lead to disease. Therefore, a comprehensive exploration of the mechanisms of cell death in heart failure is crucial for advancing treatment options and improving patient outcomes.

## 2. Definition and Classification of Programmed Cell Death

PCD is a critical biological process that plays an essential role in maintaining cellular homeostasis, development, and the elimination of damaged or diseased cells. PCD encompasses various mechanisms, including apoptosis, autophagy, necroptosis, pyroptosis, and ferroptosis; each is characterized by distinct morphological and biochemical characteristics [6]. Understanding these forms of cell death is crucial for elucidating their implications in health and disease, particularly in cancer, neurodegenerative disorders, and immune responses. Apoptosis is often considered the classical form of PCD, characterized by cell shrinkage, chromatin condensation, and DNA fragmentation, leading to the orderly removal of cells without eliciting an inflammatory response. In contrast, necroptosis is a regulated form of cell death, characterized by distinct morphological and biochemical features that distinguish it from traditional necrosis and apoptosis. The initiation of necroptosis is marked by the activation of receptor-interacting protein kinases (RIPK1 and RIPK3) and mixed lineage kinase domain-like protein (MLKL), ultimately resulting in cellular damage and inflammation [7]. Autophagy, while primarily a survival mechanism, can also lead to cell death under certain conditions. Emerging forms of PCD, such as pyroptosis and ferroptosis, have received increased attention for their roles in inflammation and the progression of cancer cells. Pyroptosis is characterized by gasdermin D (GSDMD) mediated pore formation in the plasma membrane, resulting in cell lysis and the release of pro-inflammatory cytokines. Ferroptosis, an iron-dependent form of cell death, is marked by the accumulation of lipid peroxides and is implicated in various pathological conditions, including cancer and neurodegeneration. The classification and understanding of these diverse PCD mechanisms are vital for developing targeted therapeutic strategies in various diseases. The difference between apoptosis, autophagy, necroptosis, pyroptosis and ferroptosis is shown in Table 1.

**Table 1.** **The differences between apoptosis, autophagy, necroptosis, pyroptosis and ferroptosis**.

Programmed cell death	Apoptosis	Autophagy	Necroptosis	Pyroptosis	Ferroptosis
Trigger	DNA damage, oxidative stress, viral infections, toxins, radiation, damage-associated molecules patterns (DAMPs)	Nutrient deprivation, stress, damage	TNFα, viral infection, caspase inhibition	Pathogen-associated molecular patterns (PAMPs), DAMPs	Glutathione depletion, Iron overload
Key molecules	Caspases (e.g., caspase-3, -9), BCL-2 family	ATG proteins, LC3, Beclin-1	Receptor-interacting protein kinases (RIPK1, RIPK3), mixed lineage kinase domain-like protein (MLKL)	Caspase-1, gasdermin D	GPX4, iron, lipid reactive oxygen species (ROS)
Morphological features	Cell shrinkage, chromatin condensation, DNA fragmentation	Formation of autophagosomes, vacuoles	Cell swelling, membrane rupture	Cell swelling, membrane rupture, pore formation	Cell shrinkage, Mitochondrial damage
Inflammation	Non-inflammatory (predominantly), Inflammatory (occasionally)	Non-inflammatory	Inflammatory	Highly inflammatory	Inflammatory
Outcomes	Phagocytosis of apoptotic bodies	Recycling of cellular components	Release of cellular contents	Release of pro-inflammatory cytokines	Accumulation of lipid peroxides

BCL, B-cell lymphoma; ATG, autophagy-related genes; LC3, microtubule-associated protein 1A/1B-light chain 3; GPX4, glutathione peroxidase 4.

## 3. Apoptosis in Heart Failure

### 3.1 Mechanisms and Characteristics of Apoptosis

**Fig. 1.**
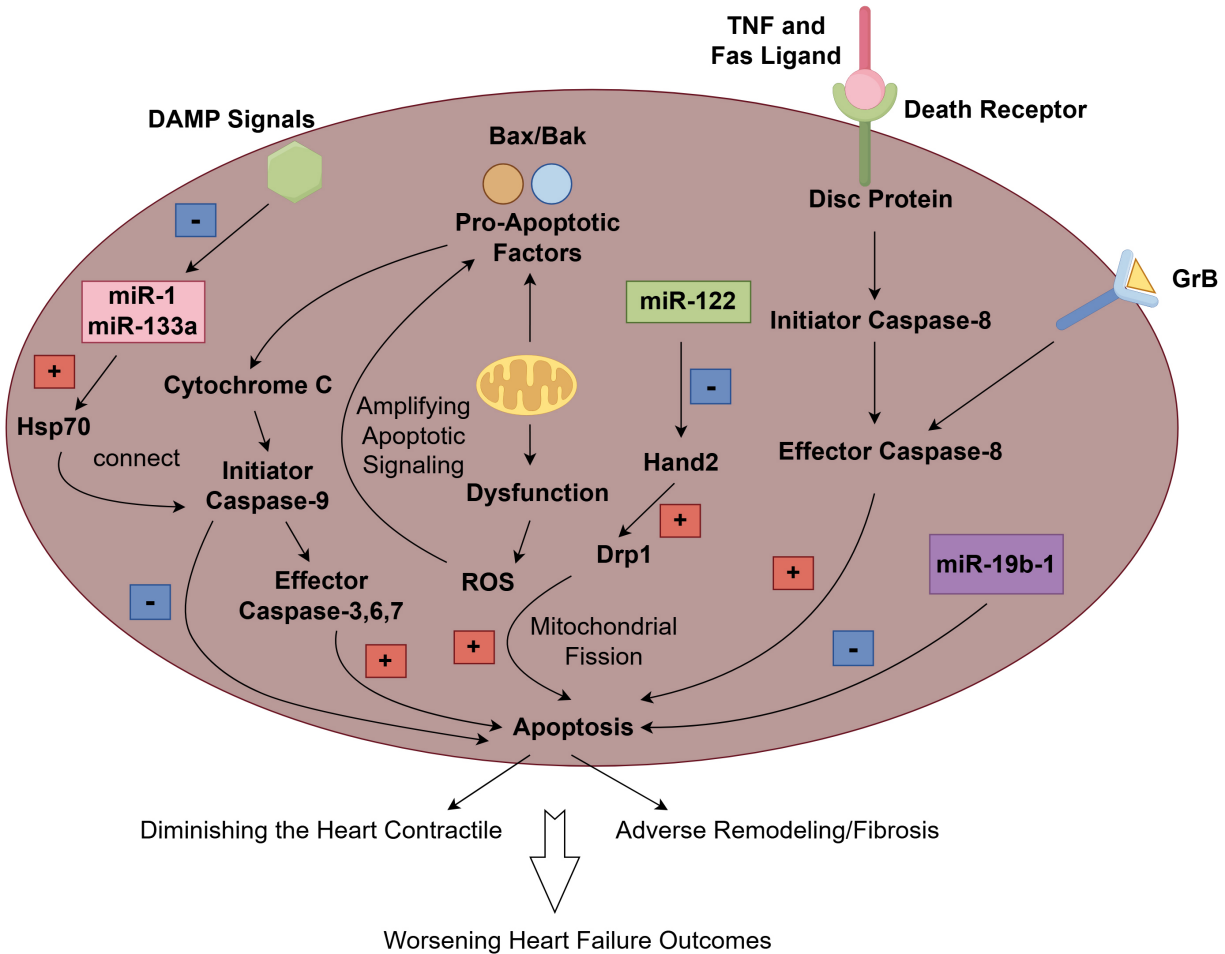
**The mechanism and role of apoptosis in heart failure**. The extrinsic pathway is initiated when death ligands (such as TNF and Fas Ligand) bind to their canonical death receptors and subsequently form the death-inducing signaling complex (DISC), which then activates caspase 8 as its effector, resulting in apoptosis. Granzyme B (GrB), as a potential multifunctional pro-inflammatory molecule, participates in the activation of caspase 8, which contributes to apoptosis. In the intrinsic pathway, pro-apoptotic proteins like Bax and Bak facilitate the release of cytochrome c from the mitochondria into the cytosol. This release triggers the activation of caspases, especially caspase-9, which subsequently activates effector caspases such as caspase-3, leading to apoptosis. Additionally, mitochondrial dysfunction frequently leads to an increased production of reactive oxygen species (ROS), which can further enhance apoptotic signaling and contribute to cell death. damage-associated molecules pattern (DAMP) signals can induce the down-regulation of miR-1 and miR-133, which in turn might facilitate the translation of crucial apoptosis regulators, such as the heat shock protein 70 (HSP70). The HSP70 chaperone is capable of connecting the apoptotic protease-activating factor 1 (APAF1)/CASPASE3 complex and caspase-9, potentially suppressing the activation of apoptosis. microRNA (miR)-122 can promote cardiomyocyte apoptosis by inhibiting the Hand2 transcription factor, which subsequently leads to an increase in the expression of dynamin-related protein-1 (Drp1). However, miR-19b-1 has been found to counteract cardiac apoptosis. The cumulative effect of increased cardiomyocyte apoptosis not only reduces the heart’s contractile capacity but also promotes adverse remodeling and fibrosis, and ultimately leads to a worsening outcome of heart failure. Fig. 1 was created with Figdraw.

Apoptosis is a highly regulated form of regulated cell death that is essential for normal development and tissue homeostasis. It is characterized by specific morphological changes, including cell shrinkage, chromatin condensation, and membrane blebbing, followed by the formation of apoptotic bodies that are phagocytosed by neighboring cells or macrophages, preventing inflammation. It involves apoptosis-related signaling pathways and the mitochondrial pathway. The mitochondrial pathway of apoptosis is primarily mediated by the intrinsic pathway, which involves the release of pro-apoptotic factors from the mitochondria. Key players in this pathway include members of the B-cell lymphoma (BCL)-2 family, which regulate mitochondrial membrane permeability. When cells are under stress or damaged, pro-apoptotic proteins such as Bax and Bak promote the release of cytochrome c from the mitochondria into the cytosol. This release activates caspases, particularly caspase-9, which subsequently activates effector caspases like caspase-3, leading to cellular disruption and death [8]. Additionally, mitochondrial dysfunction often results in increased production of reactive oxygen species (ROS), which can further amplify apoptotic signaling and contribute to cell death [9]. The interplay between mitochondrial dynamics, including fission and fusion, also plays a crucial role in determining the viability of cells during stress conditions. Mitochondrial fission, mediated by proteins such as dynamin-related protein-1 (Drp1), is often associated with the promotion of apoptosis, while fusion processes can help maintain mitochondrial function and prevent cell death [10]. The death receptor pathway, also known as the extrinsic pathway, is initiated by the binding of death ligands, such as tumor necrosis factor (TNF) and Fas ligand, to their respective receptors on the cell surface. This interaction leads to the formation of a death-inducing signaling complex (DISC), which activates caspase-8. Activated caspase-8 can directly cleave and activate downstream effector caspases, leading to apoptosis [11]. A recent study demonstrated that the activation of caspase-8 is not solely dependent on TNF but is also influenced by granzyme B (GrB) through both direct and indirect mechanisms [12]. GrB is a serine protease that is produced by a variety of immune, non-immune, and tumor cells. This enzyme has the capability to cleave components of the extracellular matrix (ECM), cytokines, cell receptors, and clotting proteins, making GrB a potential multifunctional pro-inflammatory molecule [13]. Its involvement in these processes suggests that GrB may play a significant role in the development of various inflammatory conditions. These include inflammaging, which refers to the chronic, low-grade inflammation associated with aging, as well as both acute and chronic inflammatory and cardiovascular diseases [14]. Studies have found that after an acute myocardial infarction in mice, CD8+ T lymphocytes are drawn to and activated within ischemic heart tissue. These activated lymphocytes release granzyme B, which contributes to the apoptosis of cardiomyocytes. This process ultimately results in adverse ventricular remodeling and a decline in myocardial function [15]. This pathway is particularly important in immune responses, helping to eliminate infected or damaged cells. The death receptor pathway can also trigger necroptosis, a form of regulated necrosis, when caspase-8 is inhibited [16]. The regulation of this pathway is complex and involves various post-translational modifications, such as ubiquitination, which can modulate the activity of death receptors and associated signaling proteins [17]. Apoptosis plays a critical role in the pathophysiology of heart failure (Fig. 1).

### 3.2 The Role of Apoptosis in Heart Failure

Cardiomyocyte apoptosis is a significant contributor to the loss of cardiac tissue and function, leading to HF. Studies have shown that various factors, including oxidative stress, inflammation, and neurohormonal activation, can trigger apoptosis in cardiomyocytes during heart failure [18, 19]. The cumulative effect of increased cardiomyocyte apoptosis not only diminishes the heart’s contractile capacity but also promotes adverse remodeling, fibrosis, and ultimately increases the morbidity and mortality associated with end-stage heart failure. Therefore, understanding the mechanisms of cardiomyocyte apoptosis is essential for developing targeted therapies aimed at mitigating cell death and preserving cardiac function in patients with heart failure.

When cardiomyocytes undergo apoptosis, the loss of functional cells leads to decreased contractility and impaired cardiac output. This decline in cardiac function is exacerbated by the inflammatory response that follows cell death, which further contributes to myocardial damage and remodeling. The activation of these pathways has been shown to impair cardiac remodeling resulting in decreased cardiac function in heart failure models [20]. Several studies have found that the balance between apoptosis and protective mechanisms, such as autophagy, is crucial for maintaining cardiac homeostasis. When apoptosis is dysregulated, it can lead to excessive loss of cardiomyocytes, resulting in further progression of heart failure [21, 22]. Therapeutic strategies targeting the apoptotic pathways, such as inhibiting caspase activation or promoting cell survival signals, have shown promise in preclinical studies, suggesting that modulation of regulated cell death could be a viable approach to improve cardiac function in heart failure patients [23].

The expression of apoptosis biomarkers in heart failure patients plays a critical role in understanding the underlying mechanisms of cardiac dysfunction which guide therapeutic strategies. Research has shown that various microRNAs, such as microRNA (miR)-122 and miR-19b-1, are significantly elevated in patients with heart failure. miR-122 has been identified as a key player in promoting cardiomyocyte apoptosis by inhibiting the Hand2 transcription factor, which subsequently increases the expression of Drp1, a protein associated with mitochondrial fission and apoptosis [24]. Similarly, miR-19b-1 has been found to counteract ischemia-induced cardiac apoptosis, thereby protecting cardiac function and reducing infarct size following a myocardial infarction [25]. Furthermore, studies have shown that miR1 and miR133a play a crucial role in triggering the early processes that lead to myocardial hypertrophy and early epicardial activation following an infarct [26, 27]. In the hearts of mammals and vertebrates, damage-associated molecules pattern (DAMP) signals can lead to the down-regulation of miR1 and miR133a, which may subsequently promote the translation of key apoptosis regulators, such as the heat shock protein 70 (HSP70) [27]. The HSP70 chaperone is capable of connecting the apoptotic protease-activating factor 1 (APAF1)/CASPASE3 complex and caspase-9, potentially inhibiting the activation of apoptosis [28]. Therefore, blockage of HSP70 can prevent apoptosis and attenuates cardiac remodeling and dysfunction [29]. HSP60 may also play a significant role in heart failure through this process [30]. In addition, the overactivation of β-adrenergic receptors (β-AR) in heart failure is linked to increased oxidative stress and apoptosis, highlighting the importance of mitochondrial dysfunction in the progression of heart failure [31]. These findings underscore the potential of using apoptosis biomarkers as indicators of disease severity and therapeutic targets in the treatment of heart failure.

### 3.3 New Strategies for Treating Heart Failure With Apoptosis

Pharmacological interventions targeting apoptosis are gaining traction as a vital strategy in the management of heart failure. Recent studies have highlighted the role of specific microRNAs, such as miR-122, miR-125b and miR-19b-1, in regulating cardiomyocyte apoptosis, which is a critical factor in the progression of heart failure [24, 25, 32]. These findings suggest that manipulating apoptotic pathways through pharmacological interventions could provide a novel therapeutic approach to mitigate heart failure progression and improve patient outcomes [33].

Gene therapy and cell therapy represent the forefront of innovative treatment strategies with the potential to revolutionize heart failure management. Gene therapy interventions, particularly those utilizing CRISPR/Cas9 technology, are being explored for their ability to correct genetic defects associated with heart failure and to enhance the regenerative capacity of cardiac tissues. Recent advancements in gene editing have shown promise in addressing inherited forms of heart disease, such as hypertrophic cardiomyopathy and dilated cardiomyopathy, by targeting specific mutations involved in the pathophysiology of these diseases [34]. Cellular therapies, including the transplantation of stem cells or genetically modified cells, restore cardiac function by promoting tissue regeneration and improving mechanisms involved in myocardial tissue repair. Clinical trials are currently underway to evaluate the efficacy and safety of these therapies, with early results indicating potential benefits in terms of cardiac function and patient quality of life. As research progresses, the integration of gene and cell therapies could provide a transformative approach to treating heart failure, moving beyond the management of symptoms to address the root cause of these diseases.

## 4. Autophagy in Heart Failure

### 4.1 Mechanisms and Characteristics of Autophagy

Autophagy is a vital cellular process that involves the degradation and recycling of cellular components, and plays a crucial role in maintaining cellular homeostasis. It is characterized by the formation of autophagosomes, which encapsulate damaged organelles and proteins before fusing with lysosomes for degradation. The process can be broadly classified into three types: macroautophagy, microautophagy, and chaperone-mediated autophagy (CMA) [35]. Macroautophagy involves the bulk degradation of cytoplasmic components, while microautophagy directly engulfs small portions of the cytoplasm. CMA, on the other hand, selectively degrades specific proteins that have a KFERQ-like motif, which are recognized by chaperones and transported to lysosomes for degradation. Each type of autophagy has distinct molecular mechanisms and regulatory pathways that adapt to various physiological and pathological conditions, ensuring that cells can respond effectively to stressors such as nutrient deprivation, oxidative stress, and damaged organelles [36]. The intricate regulation of autophagy is essential, as dysregulation can lead to a variety of diseases, including cancer, neurodegenerative disorders, and cardiovascular diseases, highlighting the need for a deeper understanding of its mechanisms [37].

The classification of autophagy is primarily based on the mechanism of cargo delivery to lysosomes. Macroautophagy is the most studied form, characterized by the formation of double-membrane vesicles known as autophagosomes. These vesicles engulf cellular components and subsequently fuse with lysosomes, where the contents are degraded by lysosomal enzymes. Microautophagy, in contrast, involves the direct invagination of the lysosomal membrane to engulf cytoplasmic material, while CMA selectively targets specific proteins for degradation. Autophagy is regulated by a complex network of signaling pathways and cellular factors that respond to various stressors and cellular conditions. Key regulators include the mechanistic target of rapamycin (mTOR) pathway, which serves as a central hub for nutrient sensing and energy status, inhibiting autophagy when nutrients are abundant. Conversely, under conditions of stress, such as low energy levels or hypoxia, AMP-activated protein kinase (AMPK) is activated, promoting autophagy to maintain cellular homeostasis. Transcription factors like transcription factor EB (TFEB) also play a crucial role in regulating the expression of autophagy-related genes, thereby enhancing the autophagic response during stress [38]. Other regulatory factors include various protein complexes and post-translational modifications, such as ubiquitination and phosphorylation, which modulate the activity of autophagy-related proteins. The interplay between these regulatory factors is essential for the proper functioning of autophagy. Disruptions in this regulatory network can contribute to the pathogenesis of various diseases, including cancer, neurodegenerative disorders and heart failure [39].

### 4.2 The Role of Autophagy in Heart Failure

The relationship between autophagic activity and heart failure is complex and multifaceted. Autophagy serves as a protective mechanism that helps to clear damaged cellular components, thereby promoting cardiomyocyte survival and function. In heart failure, autophagic activity can be altered, leading to either insufficient or excessive autophagy. Insufficient autophagy has been linked to the accumulation of damaged organelles and proteins, contributing to cellular dysfunction and apoptosis. Conversely, excessive autophagy can lead to autophagic cell death, further exacerbating heart failure. Research has shown that the regulation of autophagy is influenced by various factors, including oxidative stress, mitochondrial dysfunction, and the availability of nutrients, which are all prevalent in heart failure [40]. The interplay between autophagy and other cellular pathways, such as apoptosis and inflammation, complicates our understanding of its role in heart failure. For example, studies have demonstrated that the activation of autophagy can be protective in the early stages of heart failure but may become maladaptive as the disease progresses [41].

The mTOR is a central regulator of cell growth, proliferation, and autophagy. In heart failure, mTOR signaling plays a dual role, influencing both the survival and death of cardiomyocytes. Under normal physiological conditions, mTOR promotes protein synthesis and inhibits autophagy, thereby promoting cellular growth. However, in heart failure, mTOR activity can become dysregulated, leading to excessive autophagy or apoptosis. Studies have shown that inhibiting mTOR can enhance autophagic flux, which may help in the clearance of damaged organelles and proteins, thus promoting cardiomyocyte survival [42]. mTOR is influenced by various factors, including nutrient availability and cellular stress, which can further complicate its role in heart failure [21].

AMPK serves as an energy sensor in cells, responding to changes in cellular energy status. Activation of AMPK promotes autophagy, which is particularly beneficial in heart failure, where energy depletion is common. AMPK activation enhances mitochondrial biogenesis and function, thereby improving cellular energy homeostasis. Studies have shown that AMPK can inhibit mTOR signaling, creating a regulatory feedback loop that promotes autophagy while suppressing excessive cellular growth. AMPK is also involved in the response to various stressors, such as hypoxia and nutrient deprivation, which are prevalent in heart failure [43].

**Fig. 2.**
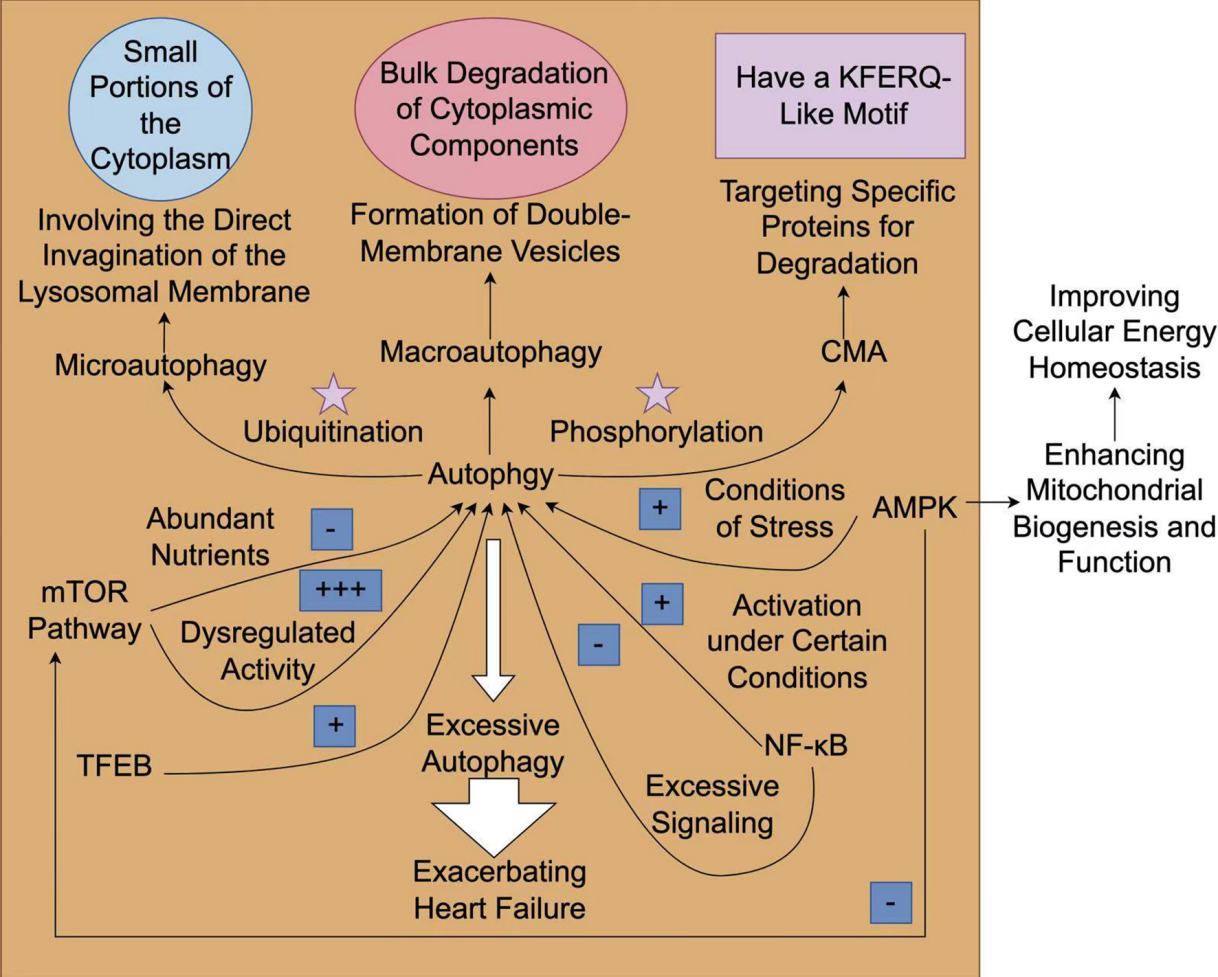
**The mechanism and role of autophagy in heart failure**. The process of autophagy can be generally classified into three types: macroautophagy, microautophagy, and chaperone-mediated autophagy (CMA). Macroautophagy is associated with the large-scale degradation of cytoplasmic components, while microautophagy directly engulfs small parts of the cytoplasm. On the contrary, CMA selectively degrades specific proteins that possess a KFERQ-like motif, which are recognized by chaperones and transported to lysosomes for degradation. Rapamycin (mTOR) signaling has a dual role in autophagy, which acts as a central hub for nutrient sensing and energy status. It inhibits autophagy when nutrients are plentiful. On the contrary, under stressful conditions, such as low energy levels or hypoxia, adenosine monophosphate-activated protein kinase (AMPK) is activated, facilitating autophagy to maintain cellular homeostasis. The activation of AMPK triggers autophagy in heart failure, which can enhance mitochondrial biogenesis and function. Transcription factors such as transcription factor EB (TFEB) enhance the autophagic response during stress. Other regulatory factors such as ubiquitination and phosphorylation modulate the activities of autophagy-related proteins. Under certain circumstances, the activation of nuclear factor kappa-light-chain-enhancer of activated B cells (NF-κB) can promote autophagy; however, excessive signaling of NF-κB may lead to autophagic dysfunction and cell death. Excessive autophagy may result in autophagic cell death, thereby further aggravating heart failure. Fig. 2 was created with Figdraw.

Nuclear factor kappa-light-chain-enhancer of activated B cells (NF-κB) is a key transcription factor that regulates inflammatory responses and cell survival. In heart failure, NF-κB signaling is often activated, leading to the expression of pro-inflammatory cytokines and contributing to myocardial inflammation and remodeling. The interplay between NF-κB and autophagy is complex. While NF-κB activation can promote autophagy under certain conditions, excessive NF-κB signaling may lead to autophagic dysfunction and cell death [44, 45]. Studies have shown that targeting NF-κB signaling can mitigate inflammation and improve cardiac function in heart failure models, suggesting that modulation of this pathway may be a viable therapeutic approach [46] (Fig. 2).

### 4.3 New Strategies for Treating Heart Failure With Autophagy

The exploration of autophagy regulators has found several various pharmacological agents that can modulate autophagic processes. Compounds such as rapamycin, which inhibits the mTOR pathway, have been shown to induce autophagy, thereby enhancing cellular resilience against stressors such as oxidative stress and inflammation. Recent studies have also identified natural products, such as curcumin and resveratrol, which can positively influence autophagy and have potential applications in treating cardiovascular diseases, including heart failure [47]. In addition, genetic approaches targeting autophagy-related genes have provided insights into the molecular pathophysiology of autophagy regulation. The interplay between microRNAs and autophagy has emerged as a critical area of study, with specific microRNAs being implicated in the modulation of autophagic activity in cardiomyocytes. As research continues to elucidate the complex signaling networks involved in autophagy, the development of targeted therapies that can effectively regulate autophagy holds promise for improving clinical outcomes in heart failure and other related conditions [40].

The therapeutic landscape for heart failure is evolving, with autophagy modulation emerging as a novel strategy. New strategies are focusing on pharmacological agents that can selectively enhance autophagy in cardiomyocytes, potentially leading to improved cardiac function and reduced morbidity associated with heart failure. For instance, the use of sodium-glucose cotransporter 2 (SGLT2) inhibitors has shown promise in enhancing autophagic activity and improving heart failure outcomes by targeting metabolic pathways that intersect with autophagy. Furthermore, ongoing clinical trials are investigating the efficacy of autophagy modulators in combination with conventional heart failure therapies, aiming to provide a synergistic effect that could lead to better patient outcomes. As our understanding of the role of autophagy in heart failure increases, these innovative strategies may offer new hope for patients suffering from this debilitating condition [48, 49].

## 5. Necroptosis in Heart Failure

### 5.1 Mechanisms and Characteristics of Necroptosis

**Fig. 3.**
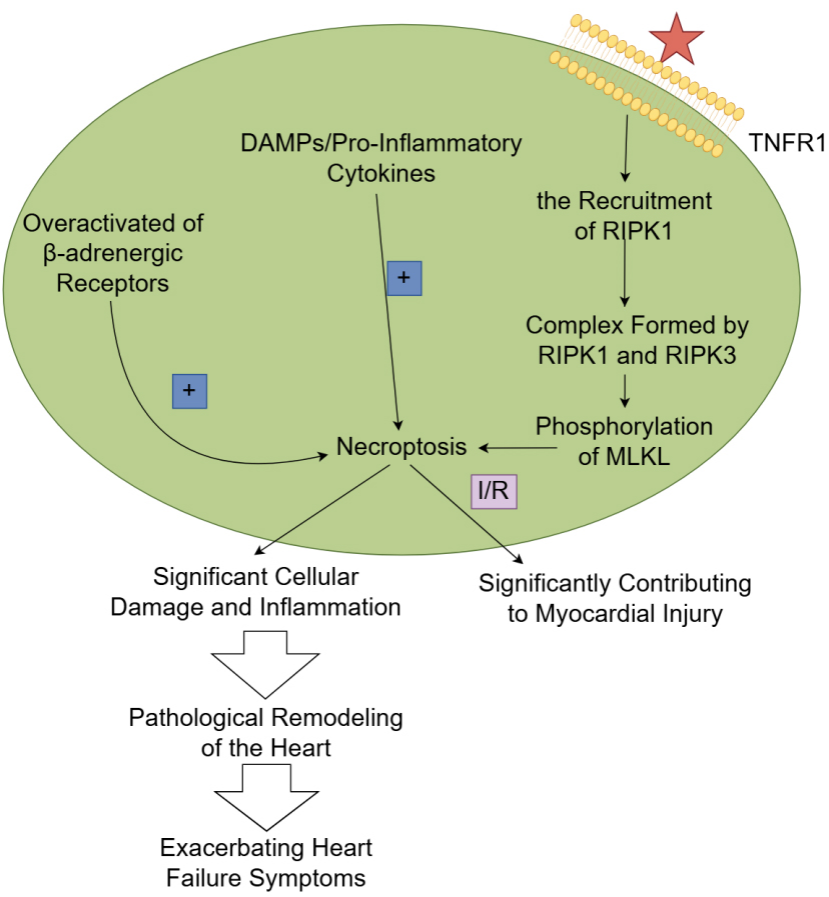
**The mechanism and role of necroptosis in heart failure**. TNF-α activates tumor necrosis factor receptor 1 (TNFR1), subsequently resulting in the recruitment of receptor-interacting protein kinases 1 (RIPK1). Once activated, RIPK1 can form a complex with receptor-interacting protein kinases 3 (RIPK3), thereby leading to the phosphorylation of mixed lineage kinase domain-like protein (MLKL). Activated MLKL oligomerizes and inserts into the plasma membrane to carry out necroptosis, ultimately leading to the rupture of the plasma membrane. When cardiomyocytes undergo necroptosis, they release damage-associated molecular patterns (DAMPs) and pro-inflammatory cytokines, thereby further intensifying inflammation and tissue damage. Overactivation of β-adrenergic receptors in cardiomyocytes contributes to necroptosis in heart failure. As necroptosis causes significant cellular damage and inflammation, it contributes to the pathological remodeling of the heart, which is characterized by fibrosis and hypertrophy, thereby further exacerbating the symptoms of heart failure. I/R, ischemia-reperfusion. Fig. 3 was created with Figdraw.

Necroptosis is a regulated form of necrotic cell death that plays a significant role in various pathological conditions, including inflammation, neurodegeneration, and cancer. It is characterized by distinct morphological changes that resemble necrosis, such as plasma membrane rupture and cytoplasmic swelling, leading to the release of intracellular contents and subsequent inflammatory responses [50]. The signaling pathways that govern necroptosis are complex and involve multiple receptors and kinases. The initiation of necroptosis typically begins with the activation of death receptors, such as tumor necrosis factor receptor 1 (TNFR1), which leads to the recruitment of RIPK1 [51]. Upon activation, RIPK1 can form a complex with RIPK3, leading to the phosphorylation of MLKL, which is crucial for the necroptotic process [52]. In addition to the classical pathway, Z-DNA binding protein 1 (ZBP1) has been identified as a crucial regulator of necroptosis, which is mainly caused by virus infection [53]. In the pathophysiology of ZBP1-mediated necroptosis, ZBP1 recruits RIPK3 via its RIP homotypic interaction motif (RHIM) domain and triggers the autophosphorylation of RIPK3. The interaction between ZBP1 and RIPK3 is sufficient to generate a distinct type of necrosome, which then leads to the phosphorylation of MLKL. In this context, RIPK1 typically functions as a negative regulator of necroptosis within the ZBP1-mediated pathway [54]. Unlike apoptosis, necroptosis does not rely on caspases; instead, it is characterized by a caspase-independent mechanism of cell death that can result in significant inflammatory responses [55]. The interplay between these signaling pathways highlights the potential for targeting specific components of the necroptotic pathway to modulate cell death, particularly in diseases characterized by excessive inflammation or cell death [56] (Fig. 3).

### 5.2 The Role of Necroptosis in Heart Failure

Necroptosis has emerged as a significant contributor to the pathophysiology of heart failure. Unlike apoptosis, which is characterized by cellular shrinkage and nuclear fragmentation, necroptosis is marked by cell swelling, membrane rupture, and the release of pro-inflammatory factors, leading to a robust inflammatory response. In the context of heart failure, necroptosis can be triggered by various stressors, including oxidative stress, calcium overload, and metabolic disturbances, often resulting from the overactivation of β-adrenergic receptors in cardiomyocytes [31]. The impact of necroptosis on cardiac function in heart failure is profound. The loss of cardiomyocytes due to necroptosis directly correlates with reduced contractility and impaired cardiac output, which are hallmark features of heart failure. The relationship between regulated necrosis and myocardial cell injury is particularly evident in the context of ischemia-reperfusion (I/R) injury, where the restoration of blood flow after a period of ischemia leads to further cellular damage. During I/R injury, necroptosis has been shown to significantly contribute to myocardial injury. Studies have shown that inhibiting necroptosis can reduce infarct size and improve cardiac function [57, 58]. The inflammatory response plays a pivotal role in mediating necroptosis in heart failure. When cardiomyocytes undergo necroptosis, they release DAMPs and pro-inflammatory cytokines, which further exacerbate inflammation and tissue damage. This inflammatory milieu not only promotes additional cardiomyocyte death but also contributes to the activation of immune cells, perpetuating a cycle of necroptosis and inflammation. As necroptosis leads to significant cellular damage and inflammation, it contributes to the pathological remodeling of the heart, characterized by fibrosis and hypertrophy, which further exacerbates heart failure symptoms [59, 60]. In summary, the interplay between necroptosis and other forms of cell death, such as apoptosis, complicates the overall picture of cardiac cell death in heart failure [61].

### 5.3 New Strategies for Treating Heart Failure With Necroptosis

Pharmacological interventions targeting the necroptosis pathway have emerged as a promising strategy for treating various diseases. Key components of the necroptosis signaling pathway include RIPK1, RIPK3 and MLKL. Inhibitors of these proteins, such as necrostatin-1, have shown efficacy in preclinical models by preventing necroptosis and reducing inflammation associated with tissue damage [52]. Studies have demonstrated that inhibiting necroptosis can improve cardiac function and reduce myocardial injury in experimental models of heart failure [62, 63].

## 6. Pyroptosis in Heart Failure

### 6.1 Mechanisms and Characteristics of Pyroptosis

**Fig. 4.**
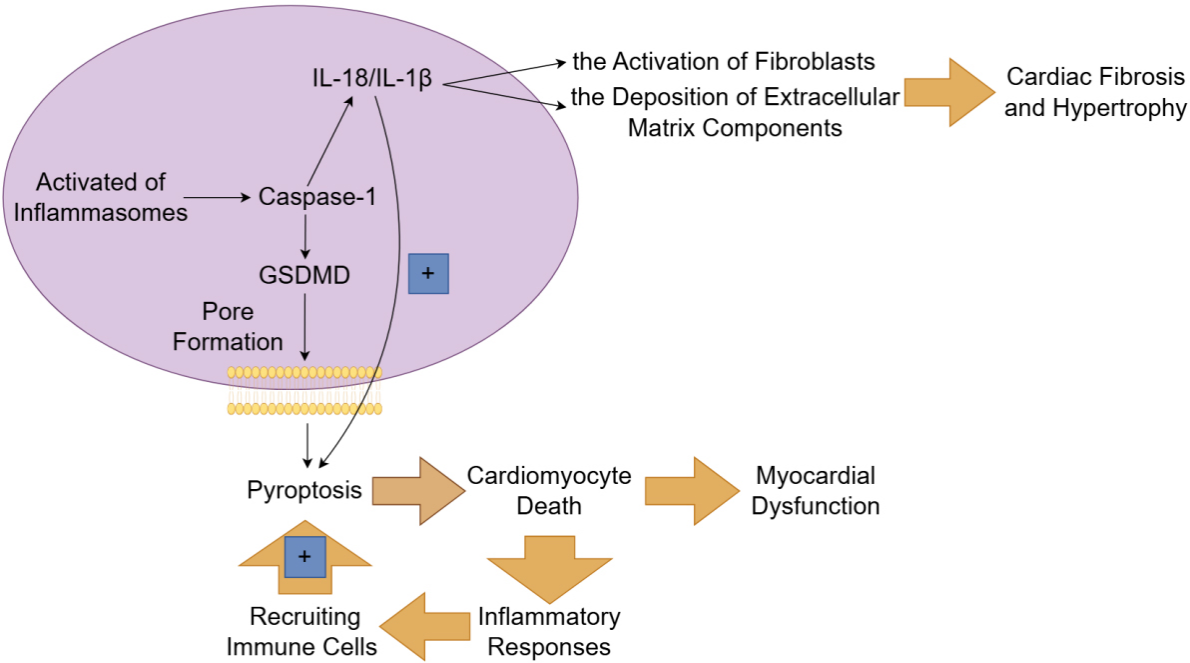
**The mechanism and role of pyroptosis in heart failure**. The canonical pathway of pyroptosis depends on caspase-1 and is initiated by the activation of inflammasomes. Once activated, inflammasomes enable the cleavage of gasdermin D (GSDMD) by caspase-1, leading to the formation of pores in the cell membrane and subsequent cell lysis. The inflammatory cytokines (such as interleukin (IL)-1β and IL-18) released during pyroptosis contribute to the activation of fibroblasts and the deposition of extracellular matrix components, thereby resulting in cardiac fibrosis and hypertrophy. Pyroptosis not only causes the loss of functional cardiomyocytes but also triggers an inflammatory response, which recruits immune cells to the injury site, perpetuating the cycle of inflammation and tissue damage. Fig. 4 was created with Figdraw.

Pyroptosis is a form of regulated cell death that is characterized by the simultaneous combination of apoptosis and necrosis. This complex process involves a series of significant changes within the cell involving nuclear condensation, organelle swelling, DNA breakage, cell membrane pore formation, and the destruction of the cell membrane [64]. The release of pro-inflammatory cytokines, including interleukin (IL)-1β, IL-18, high mobility group box-1 protein (HMGB-1), and heat shock proteins (HSPs), leads to a strong inflammatory response. These reactions can exacerbate organ dysfunction and contribute to the development of various diseases. It is primarily triggered by the activation of inflammasomes, which are multiprotein complexes that detect pathogenic microorganisms and cellular stress signals. The canonical pathway of pyroptosis is a caspase-1-dependent process triggered by the activation of inflammasomes. When pathogens invade a host cell, pattern recognition receptors (PRRs) have the ability to recognize pathogen-associated molecular patterns (PAMPs) and DAMPs intracellularly, and bind to specific ligands. Sensor proteins (such as nod-like receptor protein 3 (NLRP3), NLR-family CARD-containing protein 4 (NLRC4), or absent in melanoma 2 (AIM2)) recruit the adaptor protein apoptosis-associated speck-like protein containing a caspase recruitment domain (CARD) (ASC), which then recruits and activates procaspase-1, forming the inflammasome complex [65]. Upon activation, inflammasomes facilitate the cleavage of GSDMD by caspase-1, resulting in the formation of pores in the cell membrane and subsequent cell lysis [66]. Pyroptosis plays a crucial role in the host defense against infections, particularly in response to bacterial pathogens. However, excessive pyroptosis can contribute to tissue damage and inflammatory diseases, including sepsis and autoimmune disorders. The regulatory mechanisms governing pyroptosis, including the roles of various signaling pathways and the interplay with other forms of cell death, are currently areas of active research. Understanding pyroptosis and its implications in health and disease can provide insights into potential therapeutic strategies for controlling inflammation and improving outcomes in infectious and inflammatory diseases [67] (Fig. 4).

### 6.2 The Role of Pyroptosis in Heart Failure

Recent studies have demonstrated that pyroptosis plays a critical role in various cardiac conditions, including myocardial ischemia/reperfusion injury, myocardial infarction, and chronic heart failure. The relationship between pyroptosis and myocardial injury is well established, as pyroptosis directly contributes to cardiomyocyte death and subsequent myocardial dysfunction. Upon activation of the pyroptotic pathway, cardiomyocytes undergo a series of morphological changes, including cell swelling and membrane rupture, which culminate in the release of intracellular contents and inflammatory mediators [68]. This process not only leads to the loss of functional cardiomyocytes, but also triggers an inflammatory response that recruits immune cells to the site of injury, perpetuating the cycle of inflammation and tissue damage [69]. Studies have shown that inhibiting pyroptosis can significantly reduce myocardial injury and improve cardiac function in heart failure models [70, 71].

Pyroptosis significantly influences cardiac remodeling, a process characterized by structural and functional changes in the heart following an injury. The inflammatory cytokines released during pyroptosis contribute to the activation of fibroblasts and the deposition of extracellular matrix components, leading to cardiac fibrosis and hypertrophy [72]. This remodeling process is detrimental, as it impairs cardiac contractility and increases the risk of heart failure. Furthermore, the inflammatory environment created by pyroptotic cell death can lead to continued activation of pro-fibrotic pathways, further exacerbating adverse remodeling. Recent research indicates that interventions aimed at inhibiting pyroptosis can mitigate these remodeling effects, suggesting that targeting this form of cell death may offer a therapeutic avenue for preventing or reversing cardiac remodeling in heart failure patients [73] (Fig. 4).

### 6.3 New Strategies for Treating Heart Failure With Pyroptosis

Given the significant role of pyroptosis in the progression of heart failure, targeting this form of cell death presents a promising area for therapeutic intervention. Various strategies have been explored to inhibit pyroptosis, including the use of small molecule inhibitors that target key components of the pyroptotic pathway. Inhibitors of caspase-1 and the NLRP3 inflammasome have shown potential in reducing inflammation and improving cardiac function in preclinical models of heart failure [74]. MCC950, a specific NLRP3 inhibitor, has been shown to alleviate fibrosis and enhance cardiac function in a mouse model by suppressing early inflammatory responses after myocardial infarction [75]. When combined with rosuvastatin (RVS), MCC950 effectively suppressed the expression of NLRP3, caspase-1, interleukin-1β, and the N-terminal domains of gasdermin D. This combination also reduced serum lactate dehydrogenase (LDH) levels, enhanced cardiac systolic function, and alleviated myocardial fibrosis in mice [76]. Irisin protected cardiac function by suppressing NLRP3 and alleviating cardiomyocyte hypertrophy caused by pyroptosis [77]. Inhibition of caspase-1 reduced the incidence of cardiac fibrosis in diabetic cardiomyopathy and enhanced cardiac function by regulating miR-135b [78]. Syringaresinol (SYR) enhanced cardiac function and mitigated myocardial injury in a mouse model of sepsis-induced cardiac dysfunction by modulating the estrogen receptor (ER)/sirtuin-1 (SIRT1)/NLRP3/GSDMD signaling pathway [79]. The development of novel pharmacological agents that can selectively inhibit pyroptosis could provide new therapeutic options for patients with heart failure, particularly in cases where traditional treatments are ineffective. Furthermore, understanding the molecular mechanisms underlying pyroptosis in heart failure could lead to the identification of biomarkers for disease progression and treatment response, ultimately improving patient outcomes [73].

## 7. Ferroptosis in Heart Failure

### 7.1 Mechanisms and Characteristics of Ferroptosis

**Fig. 5.**
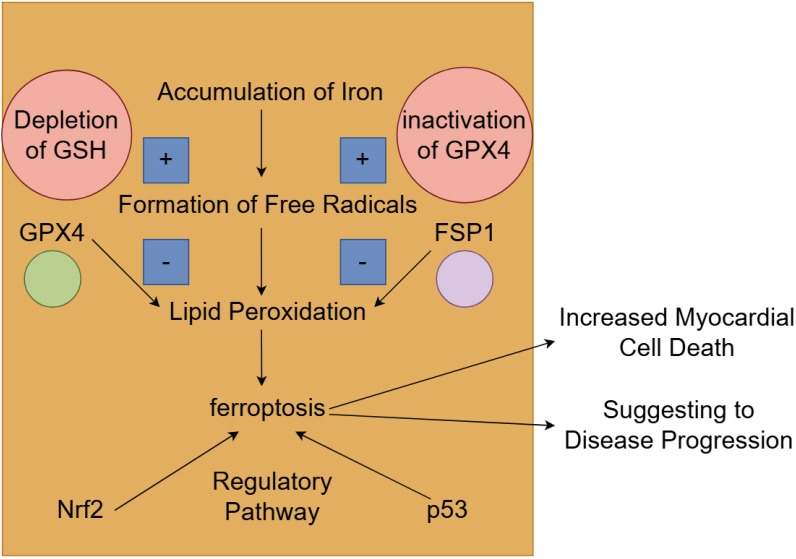
**The mechanism and role of ferroptosis in heart failure**. The accumulation of iron within cells promotes the formation of free radicals, which subsequently causes lipid peroxidation and leads to ferroptosis. This process is aggravated by the depletion of glutathione (GSH) and the inactivation of glutathione peroxidase 4 (GPX4), which usually alleviates oxidative damage. The nuclear factor erythroid 2-related factor 2 (Nrf2) and p53 signaling pathways were also involved in the regulation of ferroptosis. The activation of ferroptosis leads to increased myocardial cell death and is associated with worse clinical outcomes. FSP1, ferroptosis suppressor protein 1. Fig. 5 was created with Figdraw.

The biological characteristics of ferroptosis distinguish it from other forms of cell death, such as apoptosis and necrosis. Ferroptosis is primarily driven by the accumulation of lipid peroxides and is dependent on iron availability, which is critical for the production of ROS. The molecular mechanisms of ferroptosis are complex and involve multiple pathways that regulate iron metabolism, lipid peroxidation, and antioxidant defenses. Central to these mechanisms is the interplay between iron, ROS, and lipid metabolism [80]. The accumulation of iron within cells catalyzes the formation of free radicals, which in turn leads to lipid peroxidation. This process is exacerbated by the depletion of glutathione (GSH) and the inactivation of glutathione peroxidase 4 (GPX4), which normally mitigates oxidative damage [81]. Recent studies have identified additional regulatory pathways, including the involvement of the nuclear factor erythroid 2-related factor 2 (Nrf2) and p53 signaling pathways, which modulate ferroptosis sensitivity through their roles in antioxidant responses and iron homeostasis [82, 83]. Furthermore, the ferroptosis suppressor protein 1 (FSP1) has been recognized as a critical factor in counteracting ferroptosis by inhibiting lipid peroxidation (Fig. 5).

### 7.2 The Role of Ferroptosis in Heart Failure

Ferroptosis plays a critical role in myocardial cell injury, particularly in conditions such as diabetic cardiomyopathy and ischemia/reperfusion injury. In diabetic patients, elevated levels of intracellular lipids and iron contribute to the activation of ferroptosis, leading to increased myocardial cell death [84]. Research has demonstrated that patients with diabetic heart failure exhibit elevated markers of ferroptosis, and are associated with worse clinical outcomes [85]. The presence of ferroptosis-related proteins in heart tissue has been linked to the severity of heart failure, suggesting that ferroptosis may serve as a biomarker for disease progression [86]. Studies have shown that the inhibition of ferroptosis can significantly reduce myocardial injury and improve cardiac function, suggesting that targeting this pathway may offer a novel therapeutic approach for heart failure [87, 88]. The relationship between these proteins complicates our understanding of the various mechanisms involved in myocardial injury. For instance, the activation of ferroptosis has been linked to the loss of cardiomyocytes in various heart diseases, indicating that interventions aimed at preventing ferroptosis could preserve both myocardial integrity and function [86] (Fig. 5).

### 7.3 New Strategies for Treating Heart Failure With Ferroptosis

Traditional treatment methods for heart failure, such as angiotensin-converting enzyme (ACE) inhibitors, beta-blockers, and diuretics, primarily focus on managing symptoms and improving hemodynamics rather than addressing the underlying cellular death mechanisms. These therapies often fall short in improving long-term outcomes, particularly in patients with advanced heart failure. The limitations of current treatments are highlighted by the fact that they do not specifically target the pathways leading to cardiomyocyte death, such as ferroptosis. Studies have shown that ferroptosis contributes to myocardial injury and adverse remodeling in heart failure, indicating that targeting this pathway could provide a novel therapeutic strategy. For instance, excessive catecholamine stimulation has been linked to ferroptosis in cardiomyocytes, suggesting that interventions aimed at modulating ferroptosis could mitigate catecholamine-induced cardiac injury [89]. Furthermore, the role of ferroptosis in heart failure is supported by evidence showing that various pharmacological agents, such as SGLT2 inhibitors, can reduce ferroptosis and improve cardiac function [90]. Additionally, natural compounds such as resveratrol have been investigated for their ability to modulate ferroptosis and exert cardioprotective effects [91].

## 8. Other Programmed Cell Death Pathways in Heart Failure

Parthanatos is a type of programmed cell death that is driven by the overactivation of poly (adenosine diphosphate (ADP)-ribose) polymerase-1 (PARP-1). Unlike apoptosis, it is caspase-independent and involves the accumulation of poly (ADP-ribose) (PAR) induced by DNA damage, the release of mitochondrial apoptosis-inducing factor (AIF), and the fragmentation of nuclear DNA [92]. Conditions such as myocardial infarction, hypertension, and diabetes generate oxidative stress, damaging DNA and activating PARP-1. Chronic activation depletes NAD+/ATP, and exacerbates cardiomyocyte death [2]. Post-infarction reperfusion enhances oxidative stress, leading to the overactivation of PARP-1 and parthanatos, which contributes to the size of the infarct and ultimate the severity of cardiac dysfunction [93]. The persistent activation of PARP-1 in chronic heart failure might cause the gradual loss of cells, and is responsible for deteriorating ventricular remodeling and ultimately decreased cardiac function [94]. Preclinical studies reveal that PARP inhibitors decreased infarct size and enhanced function in animal models [95, 96]. Nevertheless, caution is needed in clinical translation because of PARP’s role in DNA repair and potential off-target effects.

NETosis constitutes a distinct form of neutrophil cell death, characterized by the release of neutrophil extracellular traps (NETs)—web-like structures composed of DNA, histones, and antimicrobial proteins. It is initiated by stimuli such as cytokines, pathogens, or tissue damage and involves peptidylarginine deiminase 4 (PAD4)-mediated chromatin relaxation [97]. In contrast to apoptosis, NETosis leads to extracellular pro-inflammatory and pro-thrombotic effects. Ischemia-reperfusion injury triggers NETosis, exacerbating inflammation, microvascular obstruction, and infarct expansion. The constituents of NETs directly cause damage to cardiomyocytes and endothelial cells [98]. Persistent immune activation in heart failure results in NETosis, which perpetuates myocardial inflammation, fibrosis, and ventricular remodeling. The elevated NET markers (such as citrullinated histones, myeloperoxidase-DNA complexes) have been correlated with the severity of heart failure. The progression of left ventricular (LV) remodeling and fibrosis at both the intermediate and late stages of HF was abolished when neutrophil depletion was achieved through either antibody-based or genetic methods [99]. NETosis plays a role in inflammation, fibrosis, and thrombosis in heart failure, thus emerging as a promising therapeutic target. Although preclinical studies have suggested deoxyribonuclease I (DNase I) and PAD4 inhibitors are potential treatments, their clinical application requires a more profound understanding of their mechanism and validation in human heart failure patients. Maintaining a balance between the inhibition of NETosis and the preservation of immune function remains a crucial challenge.

## 9. The Difference Programmed Cell Death Pathways in Diverse Etiologies of Heart Failure

In ischemic cardiomyopathy, the primary trigger is myocardial infarction, which leads to ischemia-reperfusion injury. In this case, apoptosis is likely attributed to mitochondrial damage caused by ROS and calcium overload [100]. BCL-2 proteins and caspases may also be involved. Necroptosis may also be of considerable importance, by the activation of the RIPK1/RIPK3/MLKL pathway as a consequence of TNF or other death receptors [101], or due to lipid peroxidation [102]. Inflammasomes such as NLRP3 might be activated in myocardial ischemia/reperfusion injury, leading to pyroptosis through caspase-1 and gasdermin D [103, 104]. Autophagy can be a double-edged sword—initially protective by eliminating debris, but excessive autophagy could result in cell death [105].

In non-ischemic cardiomyopathy, which encompasses elements such as genetic mutations, viral infections, or toxins, the pathways could vary. Apoptosis in this context might be initiated by mitochondrial mutations (for instance, in dilated cardiomyopathy (DCM)) or ER stress resulting from protein aggregates [106]. The administration of doxorubicin (DOX) led to the upregulation of the expressions of NADPH oxidase (NOX) 1 and NOX4. The upregulated NOX1 and NOX4 activated Drp1 and facilitated mitochondrial fission, causing excessive accumulation of ROS within mitochondria and eventually triggering NLRP3 inflammasome and caspase 1-dependent pyroptosis [107]. In addition. the treatment by DOX increased the accumulation of iron and lipid peroxidation of the membranes, thereby further leading to ferroptosis [108]. Both the activation and inhibition of autophagy flux were observed in hypertrophic cardiomyopathy (HCM) and DCM [109, 110]. Necroptosis-associated proteins, such as RIPKs and phosphorylated MLKL, were significantly upregulated in end-stage heart failure caused by DCM and heart failure resulting from myocardial infarction (MI). Phosphorylated MLKL was higher in DCM than in CAD [111]. In HCM, it is possible that pressure overload induces mechanical stress and activates necroptosis [112].

## 10. Interrelation of Different Programmed Cell Death Pathways in Heart Failure

The interrelation and complexity of different programmed cell death pathways in heart failure highlight the intricate mechanisms underlying cardiac cell loss and tissue damage. Apoptosis and autophagy frequently coexist, where autophagy initially functions as a protective mechanism but may potentially facilitate apoptosis under extreme stress [113]. Necroptosis shares upstream signaling with apoptosis. If caspase-8 is inhibited, apoptosis can be transformed into necroptosis [6]. Additionally, Ca^2+^-calmodulin-dependent protein kinase (CaMKII), which is thought to be another substrate for receptor interacting protein 3 (RIP3) in addition to MLKL, can be phosphorylated and subsequently influence mitochondrial permeability transition pore (mPTP) to regulate both necroptosis and apoptosis [114]. Furthermore, membrane permeabilization, which is mediated by the opening of the mPTP, results in both apoptosis and necroptosis [69]. RIPK1, the key mediator of necroptosis, is also involved in the modulation of autophagic signaling. Furthermore, the impaired autophagic flux promotes the activation of RIPK1 and MLKL to affect necroptosis [115]. Pyroptosis and necroptosis both increase inflammation, creating a vicious cycle of cell death. Autophagy can promote ferroptosis by degrading ferritin and increasing iron levels [116]. The cumulative effect of multiple PCD pathways leads to fibrosis, hypertrophy, and impaired cardiac function.

## 11. Limitations of Programmed Cell Death-Target Therapies

Despite the advancements in targeted therapies for programmed cell death, several limitations persist that hinder their widespread clinical application. One major challenge lies in the complexity and interconnectivity of these pathways. The cross-talk between different PCD pathways complicates the targeting process. For instance, the inhibition of caspase-8 may drive necroptosis via RIPK1/RIPK3, thereby causing inflammation. Additionally, toxicity and off-target effects can restrict their application in specific patient groups. PCD pathways sustain homeostasis in healthy tissues (such as the intestinal epithelium and immune cells). Off-target apoptosis may lead to myelosuppression or gastrointestinal toxicity. Furthermore, drug resistance can impact the therapeutic effect. Studies reveal that cancer cells evade apoptosis by upregulating anti-apoptotic proteins (such as BCL-2, myeloid cell leukemia (MCL)-1) or mutating pro-death signals like tumor protein 53 (TP53) [117]. Resistance to BCL-2 inhibitors (for instance, venetoclax) is common due to compensatory overexpression of MCL-1 [118]. Finally, the heterogeneity among patients can result in variable responses to targeted therapies. For example, although immune checkpoint inhibitors have achieved remarkable success in some patients, a considerable proportion do not respond or develop resistance over time, emphasizing the necessity of predictive biomarkers to identify those patients who are most likely to benefit from these treatments [119].

Excessive inhibition of PCD pathways could compromise physiological cell turnover or immune responses. For example, impaired apoptosis allows damaged or mutated cells to survive, promoting tumorigenesis and genomic instability [120]. Failure to eliminate autoreactive lymphocytes due to impaired apoptosis leads to diseases such as systemic lupus erythematosus (SLE). Uneliminated apoptotic cells release nuclear antigens, triggering autoantibodies [121]. In summary, PCD pathways require tight regulation. Over-inhibition disrupts homeostasis, emphasizing the need for balanced therapeutic strategies to maintain cellular health and prevent disease.

## 12. Conclusion

The role of regulated cell death in the onset and progression of heart failure is increasingly recognized as a pivotal factor influencing patient outcomes. This review has explored the intricate mechanisms of apoptosis, autophagy, necroptosis, pyroptosis, and ferroptosis shedding light on how these processes interconnect and contribute to the pathophysiology of heart failure. By better understanding these mechanisms, we can pave the way for novel therapeutic strategies that not only target individual pathways but also consider the holistic interactions between them.

Future research should prioritize the exploration of the crosstalk between these cell death modalities, aiming to decipher how they collectively influence cardiac health and disease. Understanding these interactions may reveal synergistic effects that could be channeled into therapeutic interventions. For instance, strategies that can modulate autophagic processes alongside apoptosis may offer a more comprehensive approach to improving heart function and patient prognosis.

The clinical application of targeted therapies against specific cell death pathways remains an urgent need. As we advance our understanding of these mechanisms, it is crucial to translate this knowledge into practical, evidence-based treatments that can be integrated into existing heart failure management protocols. This approach not only holds the promise of improving patient outcomes but also enhances our ability to customize therapies based on individual patient profiles.

In conclusion, the ongoing investigation into regulated cell death mechanisms offers a promising frontier in the treatment of heart failure. By combining different research perspectives and findings, we can develop innovative strategies that leverage the complexity of these processes, ultimately leading to improved prognostic and therapeutic outcomes for heart failure patients.
